# Beyond the surface: how *ex-vivo* diffusion-weighted imaging reveals large animal brain microstructure and connectivity

**DOI:** 10.3389/fnins.2024.1411982

**Published:** 2024-06-26

**Authors:** Mehdi Behroozi, Jean-Marie Graïc, Tommaso Gerussi

**Affiliations:** ^1^Department of Biopsychology, Faculty of Psychology, Institute of Cognitive Neuroscience, Ruhr-University Bochum, Bochum, Germany; ^2^Department of Comparative Biomedicine and Food Science (BCA), University of Padova, Legnaro, Italy; ^3^Department of Infectious Diseases and Public Health, Jockey Club College of Veterinary Medicine and Life Sciences, City University of Hong Kong, Hong Kong, Hong Kong SAR, China

**Keywords:** DWI, DTI, CSD, tractography, large brain, post-mortem imaging

## Abstract

Diffusion-weighted Imaging (DWI) is an effective and state-of-the-art neuroimaging method that non-invasively reveals the microstructure and connectivity of tissues. Recently, novel applications of the DWI technique in studying large brains through *ex-vivo* imaging enabled researchers to gain insights into the complex neural architecture in different species such as those of *Perissodactyla* (e.g., horses and rhinos), *Artiodactyla* (e.g., bovids, swines, and cetaceans), and *Carnivora* (e.g., felids, canids, and pinnipeds). Classical *in-vivo* tract-tracing methods are usually considered unsuitable for ethical and practical reasons, in large animals or protected species. *Ex-vivo* DWI-based tractography offers the chance to examine the microstructure and connectivity of formalin-fixed tissues with scan times and precision that is not feasible *in-vivo*. This paper explores DWI’s application to *ex-vivo* brains of large animals, highlighting the unique insights it offers into the structure of sometimes phylogenetically different neural networks, the connectivity of white matter tracts, and comparative evolutionary adaptations. Here, we also summarize the challenges, concerns, and perspectives of *ex-vivo* DWI that will shape the future of the field in large brains.

## Introduction

Recent advances in magnetic resonance imaging (MRI) have made it a powerful tool for studying brain anatomy and function in different animals ranging from humans (Pfefferbaum et al., 2004; Dawe et al., 2009; McNab et al., 2009), non-human primates (D’Arceuil et al., 2007), rodents (Zhang J. et al., 2012; Russo et al., 2021), birds (Behroozi et al., 2020; Hamaide et al., 2020; Orije et al., 2021), reptiles (Behroozi et al., 2018; Billings et al., 2020), and even marine mammals (Berns et al., 2015; Gerussi et al., 2023, 2024). Diffusion-weighted imaging (DWI) distinguishes out from other approaches because it can non-invasively analyze neuroanatomical connectivity and white matter microstructure via measuring the restricted diffusion of water molecules in tissue (Le Bihan, 1996; Assaf et al., 2019). Given that water makes up around 60–70% of the body, understanding diffusion is critical. Diffusion refers to the random, thermally-driven, Brownian motion of molecules (Mukherjee et al., 2008). Water diffusivity in a homogeneous medium occurs randomly and isotropically with equal probability in all directions. On the other hand, water diffusivity in restricted environments surrounded by walls or membranes is anisotropic, and thus the probability of diffusion is higher along one direction over the others. It is therefore the case in brain tissue where water in the extracellular space or inside the somata diffuses isotropically, while water molecules inside neuronal axons are narrowly limited by axonal membranes, and thus the probability of diffusion parallel to the major axes is higher than the perpendicular (Moseley et al., 1991; Baliyan et al., 2016).

This random Brownian motion can be accurately estimated through DWI-MR sequences, employing gradients at different high amplitudes and combinations, i.e., directions. These sequences basically measure the anisotropic characteristics of water molecules which can reflect either the normal condition of the brain parenchyma or alterations directly from the raw data (anisotropy) in case of some pathologies (Benjamin et al., 2017; Mallon et al., 2020; Makada and Matang, 2023). The development of the algorithms encoding DWI sequences can then offer insights into the micro-architectural details of white matter at each voxel. Consequently, fiber tractography enables the elucidation of white matter integrity and the delineation of axonal tracts throughout the brain.

While *in-vivo* DWI has revolutionized the study of brain connectivity, *ex-vivo* DWI approaches offer distinct advantages, particularly in: (i) *enhanced resolution: ex-vivo* imaging allows for significantly higher voxel resolutions with enhanced signal-to-noise ratios (SNR), often reaching scales of hundreds of micrometers (Roebroeck et al., 2019). This level of resolution surpasses the constraints of *in-vivo* scans, typically limited to a range of 1–3 millimeters. The finer granularity of *ex-vivo* imaging facilitates an accurate mapping of intricate neural pathways and structures. Recent advances in DWI approaches have focused on optimizing SNR, voxel resolution, MR sequence, and the capacity to image big whole brains. These developments include the use of stronger gradients, higher magnetic field strengths, and customized pulse sequences that improve the contrast and resolution of DWI images, allowing the observation of previously undetectable microstructural features. (ii) *Exploring rare and extinct species*: *ex-vivo* DWI provides an invaluable tool to study the brains of rare, endangered, and “extinct in the wild” animals. Specimens preserved in brain banks, museums, or available from veterinary services after the natural death of zoological park specimens offer researchers a unique opportunity to delve into the neural architecture of species outside of current neuroanatomical models. (iii) *Investigating large brains*: some animals possess brains and bodies that often exceed the dimensions accommodated by current *in-vivo* imaging technology. One of the main challenges lies in the absence of coils large enough to adequately capture these dimensions from the animal’s head. *Ex-vivo* approaches bypass this limitation by allowing the study of large-brain species without spatial constraints, through extraction of the brain or section of the head. This capability is particularly advantageous to investigate the neural basis of complex cognitive abilities and behavioral repertoires in species with expansive brain sizes or extreme habitats, such as cetaceans (Gerussi et al., 2023) and elephants (Hakeem et al., 2005).

This review aims to highlight the indispensable role of *ex-vivo* DWI in studying the structural architecture of the brain. By employing this technique, researchers can uncover patterns of brain organization and connectivity, shedding light on evolutionary adaptations, behavioral complexities, and ecological dynamics. *Ex-vivo* DWI thus emerges as an essential tool integrating advanced imaging methodologies to potentiate ambitious comparative neuroanatomy, furthering our understanding of brain connectivity.

## Assessing white matter connectivity

**FIGURE 1:**
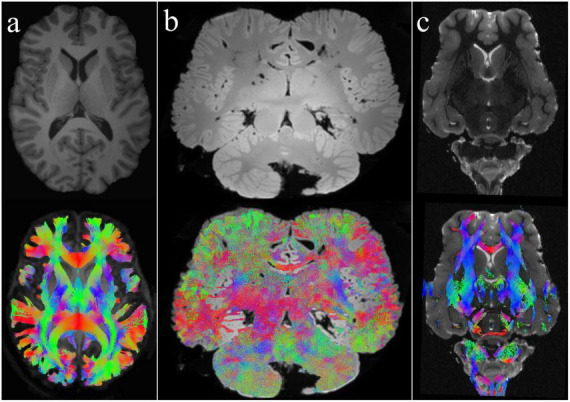
Whole-brain tractography in different species using CSD and DTI methods. **(a)** Human brain: This image represents the classical in-vivo T1-weighted structural image and the whole-brain tractography of a human brain shown here as a comparison with the other animals. The tractogram was created using CSD. **(b)** Bottlenose dolphin brain: Depicted here is an ex-vivo T2-weighted brain of a bottlenose dolphin along with its whole-brain tractogram, generated using the CSD method. **(c)** Sheep brain: The top image shows a T2-weighted anatomical view of an ex-vivo sheep brain, while the bottom image presents its corresponding whole-brain tractogram employing DTI. For more details, refer to Gerussi et al. (2022, 2023, 2024) and Graïc et al. (2023).

Fiber tractography algorithms use data from DWI scans to predict the pathways of white matter tracts based on the directional diffusion of water molecules. This method gives crucial insights into the brain’s structural connectivity, allowing researchers to investigate the structure and integrity of neuronal circuits. At the moment, there are two main physics-based approaches to modeling white matter diffusion anisotropy (i) diffusion tensor imaging (DTI) (Basser et al., 1994), and (ii) constrained spherical deconvolution (CSD) (Tournier et al., 2007). DTI analyses fiber orientation through voxel-wise evaluations (voxel refers to the smallest analytic unit), generating a singular tensor model that estimates a single three-dimensional orientation per voxel (Basser et al., 1994; Basser, 1995). This method demands minimal data points (a minimum of six independent measurements in different directions) for estimation, making it computationally efficient. While this approach is robust when there is a clear dominating diffusion direction within a voxel, it loses sensitivity when faced with multiple fiber pathways, such as crossing or branching fibers (Basser et al., 2000). In contrast, CSD as a tensor-free modeling approach, estimates the fiber orientation distribution (FOD) response function (Tournier et al., 2007, 2012) within each voxel, revealing the orientations and contributions of different fiber populations to observed diffusion behavior, and therefore does not assume isotropic diffusion (Farquharson et al., 2013). FOD does not lose information by averaging to obtain a single tensor, allowing for multiple fiber orientations to be identified. In contrast to the CSD, the simplified version of the Ball-and-Stick model proposed by Yang et al. (2019) offers notable advantages in terms of computing efficiency and implementation simplicity. While it is less intricate compared to CSD, this model operates effectively by splitting the diffusion-weighted MR signal within each voxel into multiple anisotropic components, representing different fiber orientations, along with a single isotropic component. This simplicity not only decreases processing needs but also allows for quicker implementation, making it especially helpful in cases where computational resources are restricted or swift analysis is necessary. The choice of method is determined by the individual research needs, the complexity of the fiber topologies under consideration, and the computational resources (Figure 1).

In this mini-review, we focused on physical-based diffusion models, specifically DTI and CSD, due to their established clinical use, computational efficiency, and robustness to noise (Tournier et al., 2007). These models require less stringent data acquisition parameters, making them suitable for studies with limited scanning time or lower magnetic field strengths. However, other algorithms have been developed to overcome the drawbacks of DTI (or CSD) such as Diffusion Spectrum Imaging (DSI) (Wedeen et al., 2012), Ball-and-Stick model (Yang et al., 2019), and Q-ball imaging (QBI) (Tuch, 2004). As the community seeks to gain a deeper knowledge of neuronal connections, it must look beyond DTI and tensor-free methods such as CSD to accurately model diffusion behavior furthering our understanding of brain anatomy and function. To this aim, we emphasize the potential of biophysical models like Neurite Orientation Dispersion and Density Imaging (NODDI) (Zhang H. et al., 2012) and Composite Hindered and Restricted Model of Diffusion (CHARMED) (Assaf and Basser, 2005) to provide more nuanced insights into tissue composition. However, their application is often constrained by the need for high-quality, multi-shell diffusion data, greater computational resources, and acquisition protocols that must support the model’s assumptions (Jelescu et al., 2020). We believe that future studies with access to advanced imaging modalities may benefit from incorporating biophysical models to further elucidate the complexities of neural tissue.

From DWI data, fiber pathways can be reconstructed using various models tailored to the research requirements and objectives, such as probabilistic vs. deterministic models or ROI analysis vs. whole-brain tractography [for details, see (Jeurissen et al., 2019; Zhang et al., 2022)]. Once fiber pathways have been reconstructed, quantitative assessment of specific tracts can reveal important insights into brain anatomy and function. This assessment requires examining several metrics generated from tractography data to evaluate parameters such as total tract volume, total length of reconstructed fiber tract, curvature, and connectivity strength. However, quantitative assessment of selected tracks using diffusion tensor metrics can provide information about white matter microstructural integrity. Fractional Anisotropy (FA) is a widely used parameter that measures the degree of directionality of water diffusion within a voxel and is sensitive to changes in white matter microstructure, such as myelination, crossing fiber orientation, and axonal density (Beaulieu, 2002). Furthermore, axial (AD) and radial (RD) diffusivities are the water diffusion coefficients parallel and perpendicular to the axons indicating axonal integrity and fiber myelination, respectively (Tournier et al., 2012). Furthermore, recent studies have demonstrated the potential of relaxometry parameters, such as effective transverse relaxation rate (R2*) and longitudinal relaxation rate (R1), as they provide additional information regarding tissue microstructure and composition. R2* is sensitive to iron concentration and myelin integrity (Straub et al., 2020), which are critical to the proper functioning of neural fibers. R1 values provide information on the longitudinal relaxation properties of tissue, which can indicate water content and macromolecule presence (Weiskopf et al., 2013). Understanding the fiber-specific relaxometry properties can offer enhanced specificity and sensitivity in differentiating the white matter architecture, especially in regions with crossing fibers (De Santis et al., 2016). Overall, a quantitative evaluation of selected tracts enables the characterization of white matter pathways in terms of size, shape, symmetry, and microstructural features, which is essential to understanding brain connections.

## Tissue properties and preparation

Fixed samples are frequently employed in *ex-vivo* tissue imaging because they provide the longitudinal stability necessary for longer scanning sessions. There are significant factors and trade-offs to consider when scanning *ex-vivo* tissues compared to *in-vivo*.

While fixed tissue samples present certain challenges (see below), they offer the distinct advantage of temporal stability, facilitating time-dependent enhancements in image resolution that enable meticulous analysis of structures at the micron scale. Such precision would be nearly impossible to reach in current scanning times *in-vivo*. The absence of rhythmic blood flow, and virtually any movement allows for any sequence length to be performed to ideal standards, in as many diffusion directions as desired, optimizing results while reducing artifacts. This also implies that nearly identical scans can be made repeatedly over long periods of time, on more modern machines, in bores that would not fit a whole head, or after transport from a remote location. Once the sample has been extracted and fixed, it can be kept safely available in a bank for years without significant deterioration, thus providing any researcher with brains from species they would have no access to otherwise. To be also considered is the absence of a medical or veterinary care team to monitor anesthetized *in-vivo* subjects, or trained animals in rare cases (Andics and Miklósi, 2018), and therefore the absence of risk for them, the subject, or the machine. Even in the best case, most large species are very challenging to scan because of physical, medical, and time constraints. In practical terms, an *ex-vivo* specimen is brought to room temperature, scanned, and removed at will, depending on the scanning procedure, and not the other way around.

The main issues usually reported with ex-vivo scanning regard the *post-mortem* interval (PMI, the time elapsed between death and tissue fixation) and the correct fixation of the brain. Given that tissue fixation processes either halt or significantly slow down metabolic decay, it is crucial to consider the PMI. A fundamental difference between large brains and those of small animals lies in the methods of brain tissue fixation and preservation. In small, laboratory animals, perfusion fixation via *pre-mortem* intracardiac injection of fixative, in addition to immersion fixation post-tissue extraction, reduces PMI to essentially zero. Conversely, in large animals, usually not kept in laboratory conditions, *post-mortem* perfusion fixation via mechanical pumping of fixative through the cadaver, often utilizing the femoral artery in humans or the carotid arteries and jugular veins in animals, results in a poorer diffusion of the fixative due to post-mortem blood clotting, amounting to a longer PMI (McFadden et al., 2019). In most studies on human brains, a “good” PMI is considered < 24 h (Maranzano et al., 2020). Moreover, mechanical perfusion fixation is not commonly used in larger brains due to the difficulty in washing blood clots and the possibility of microscopic damage from pumping pressure. Consequently, large brains are typically fixed via immersion. However, immersion fixation proves less effective for large brains compared to small animals due to the prolonged duration required for passive diffusion of fixative over greater distances (Dawe et al., 2009), resulting in lower-quality fixation (Werner et al., 2000; Kasukurthi et al., 2009; Gage et al., 2012). An optimized fixation protocol, taking into account all scenarios, would require the shortest PMI possible, refrigeration (but absolutely no freezing), vascular clearing using iso-osmotic solutions, then fixative perfusion and immersion. The fixative volume should be 10 times the brain volume and changed 24 to 48 h after immersion. There should be clarity on the facts that paraformaldehyde is a solid, which forms formaldehyde (a gas) in solution, and that a 4% formaldehyde solution is equal to a 10% formalin solution, with the exception that formalin contains methanol (about 1% in a 10% formalin solution) to prevent precipitation into paraformaldehyde, but which can alter immunohistochemical properties of the tissue in the long term.

Nonetheless, the use of fixatives such as formalin or other formaldehyde solutions is known to alter tissue diffusion characteristics by promoting intermolecular cross-linking (Pfefferbaum et al., 2004; Shepherd et al., 2009). Furthermore, fixed samples frequently have lower proton density and shorter T1 and T2 relaxation times than living subjects, which can be attributed to the combined effects of tissue fixation, post-mortem changes, and a lower temperature (typically room temperature, approximately 23°C) than the body’s normal temperature (Roebroeck et al., 2019). The shortened T2 relaxation time and diffusivity of fixed tissue have a considerable impact on the contrast-to-noise ratio in DWI, which is critical for high-quality *ex-vivo* DWI. To provide appropriate contrast with reduced diffusivity, higher diffusion weighting is required. Increased diffusion weighting often necessitates longer echo times, making signal loss from T2 relaxation time a limiting issue, especially when short T2 causes the unweighted-diffusion signal to decay quickly. To keep a good SNR, a possible solution could be the use of optimized sequences with shorter echo times while still keeping long diffusion times such as stimulated echo-acquisition mode [STEAM, (Merboldt et al., 1991; Rane et al., 2010)] or sequences which accumulate magnetization over multiple repetition times still keeping strong diffusion weighting such as steady-state free precession [SSFP, (McNab and Miller, 2010)]. Fixation by immersion revealed a notable decrease in T1, T2 relaxation times, and diffusivity within post-mortem brain tissue. For instance, Shepherd et al. (2009) discovered that rat cortical slices immersion-fixed in 4% paraformaldehyde had a 21% decrease in T1 and an 81% decrease in T2 compared to fresh, unfixed post-mortem values (Shepherd et al., 2009). Similar results were seen in *ex vivo* human white matter, where T1 and T2 relaxation times were measured at 340 ms and 45 ms, respectively, at 3 Tesla. These findings suggest reductions of approximately 35% in T1 and 60% in T2 (McNab et al., 2009; Miller et al., 2012). These studies show that a significant percentage of T2 drop may be attributable simply to the fixation process, which is likely caused by a combination of dehydration and the presence of unbound fixative. The decrease in T1 is integral to tissue fixation as it is predominantly influenced by the size of molecules within the tissue environment and cannot be reversed during rehydration. This reduction is likely attributed to the cross-linking of proteins, which is irreversible (Tovi and Ericsson, 1992; Shepherd et al., 2009). However, prolonged washing in saline or phosphate-buffered saline (PBS) to remove fixative and rehydrate the specimen has been shown to fully recover T2 decreases to levels equivalent to fresh, unfixed *post-mortem* samples [but not to *in-vivo* levels (Shepherd et al., 2009)].

In terms of the reduction in diffusivity observed in fixed samples, animal studies have shown a significant decrease, with a 50% reduction in apparent diffusion coefficients (ADC) observed in unfixed *ex vivo* white matter (WM) and an 80% reduction in fixed WM compared to *in-vivo* values (D’Arceuil et al., 2007). Additionally, washing fixed tissue in PBS has been demonstrated to partially reverse these effects, with reported increases in WM ADC by approximately 30%. Similar findings were observed in *ex-vivo* human brains, with WM diffusivity reduced by approximately 85% compared to *in-vivo* values (McNab et al., 2009; Foxley et al., 2014). The decreased temperature of *ex-vivo* tissues during scanning is a crucial factor contributing to the overall reduction in diffusivity. Le Bihan (1996) previously illustrated that a drop in tissue temperature by 1°C corresponds to an expected 2.4% decrease in water diffusivity. However, recent research has indicated that even when temperature adjustments are made in a manner akin to Le Bihan’s technique, differences in diffusivity persist (Dyrby et al., 2011; Walker et al., 2019). Controlling these factors using for example 40°C PBS could help alleviate part of the issue.

It should be noted that all existing challenges do not invalidate the utility and need of fixed tissue samples for studies concerning precise tissue cytoarchitecture or white matter connectivity. On the contrary, we aim to highlight the potential translational significance of fixed tissue as diffusion imaging gains prominence in examining neural microstructure and connectivity. However, when *ex-vivo* DWI-MR studies are validated against histology or compared to *in-vivo* DWI, it is necessary to consider all the challenges mentioned earlier.

## Application of *ex-vivo* large brain DWI

DWI is a neuroimaging tool with very high potential and yet little used in large brains, from domestic to wild animals. In the field of Veterinary Medicine, DWI has emerged as a valuable tool for neuropathological diagnosis in domestic animals, especially dogs and cats. It facilitates the identification of brain injuries after hypoxic lesions (Davis et al., 1994; McConnell et al., 2005; Garosi et al., 2006; Kang et al., 2009; Crawford et al., 2023), brain oedema (Zhao et al., 2006) and epileptic seizures (Hasegawa et al., 2003). Furthermore, it has been also used to investigate the normal brain anatomy and neural connectivity patterns in live cats and dogs, as well as postmortem examinations of their brains (Hasegawa et al., 2003; Ronen et al., 2003; Shaibani et al., 2006; Takahashi et al., 2010, 2011; Jacqmot et al., 2013, 2017, 2020; Gray-Edwards et al., 2014; Anaya García et al., 2015; Dai et al., 2016; Robinson et al., 2016; Das and Takahashi, 2018; Johnson et al., 2020; Andrews et al., 2022), pig (Conrad et al., 2012; Schmidt et al., 2012), bovine (Schmidt et al., 2009), and horses (Schmidt et al., 2019; Boucher et al., 2020).

In other domestic species, which emerged recently as models for translational medicine, DWI has also been adopted. It is the case of the sheep (Ovis aries) (Förschler et al., 2007; Lee et al., 2015; Peruffo et al., 2019; O’Connell et al., 2021; Pirone et al., 2021; Banstola and Reynolds, 2022; Gerussi et al., 2022; Graïc et al., 2023), the Göttingen (Knösche et al., 2015) and Yucatan (Wang et al., 2023) minipigs (*Sus scrofa*). It is worth noting that in these cases, most of the analyses were conducted using fixed brains. When compared to other laboratory animals, the brains of large ungulates such as horses, pigs, and domestic ruminants have received relatively little attention, despite their high availability following slaughter (Cozzi et al., 2020). In fact, these brains could potentially serve as a non-invasive source for the study of comparative and evolutionary neuroscience.

In the context of wild and exotic species, DWI analysis involves the study of both pathological conditions (Cook et al., 2018) and anatomical features (Cook et al., 2021; Cook and Berns, 2022) in pinnipeds but there are also investigations in one-humped camel (*Camelus dromedarius*) (Cartiaux et al., 2023) and dolphin brains (Berns et al., 2015; Wright et al., 2018; Orekhova et al., 2022; Gerussi et al., 2023, 2024). Table 1 summarizes the most important publications concerning the use of DWI in large brains.

**TABLE 1:** Bibliographic references on DWI scans of large brains with correspondent MRI sequences and DWI algorithms used.

Animal models	Type of scan	MRI machine	Sequence	Tractography technique	References and main findings
Pinnipeds	*Ex-vivo*	Siemens Healthcare, Trio, 3T	Diffusion-weighted steady-state free precession (DW-SSFP)	DTI	(Cook et al., 2018): California sea lions exposed to the algal neurotoxin domoic acid are natural animal models for human epilepsy.
	*Ex-vivo*	Siemens Healthcare, Trio, 3T	DW-SSFP	DTI	(Cook and Berns, 2022): Pinnipeds and coyotes have exceptionally larger caudate nuclei compared to putamen.
	*In-vivo*	Siemens Healthcare, Trio, 3T	DW-SSFP	DTI	(Cook et al., 2021): Established an MRI protocol for comprehensive evaluation of domoic acid toxicosis in the sea lion brain.
Horse	*Ex-vivo*	Philips Healthcare, Achieva, 3T	Spin-echo echo-planar imaging (SE-EPI)	DTI	(Boucher et al., 2020): Confirmed the presence of fiber bundles corresponding to pyramidal tracts from the motor cortex to the central brainstem.
Sheep	*In-vivo*	General Electric Healthcare, 3T	SE-EPI	DTI	(Lee et al., 2015): Demonstration of feasibility of combining DTI and fMRI in alive and healthy animals. Examples were the sensorimotor and visual cortex
	*Ex-vivo*	Bruker, 4.7T	SE-EPI	DTI	(Peruffo et al., 2019): Confirmed the presence of fiber bundles corresponding to pyramidal tracts from the motor cortex to the central brainstem. (Pirone et al., 2021): Found ipsilateral connections between the claustrum and the visual cortex. (Gerussi et al., 2022): Found a similarity in the connections of the orbitofrontal cortex between the sheep and humans, including a right asymmetry. (Graïc et al., 2023): Studied the connections of the amygdala, showing some similarities with the well-studied cat, rat, and monkey.
	*In-vivo*	Philips Healthcare, Achieva, 1.5T	SE-EPI	DTI	(Pieri et al., 2019): Created a DTI atlas of the major fiber bundles.
	*In-vivo*	Siemens Healthcare, Skyra, 3T	SE-EPI	DTI	(O’Connell et al., 2021): Used DTI and magnetic resonance spectroscopy in the striatum to study the excitotoxic model of Huntington disease.
Pig	*Ex-vivo*	Varian, 4.7T	Pulse gradient spin echo (PGSE)	DTI and CSD	(Knösche et al., 2015): Found that various DWI protocols are likely to find major fiber bundles but are less sensitive to complex fiber architecture and high spatial resolution and SNR are needed.
	*In-vivo* and *Ex-vivo*	Philips Healthcare, Achieva, 1.5T	SE-EPI	DTI and CSD	(Walker et al., 2019): Compared the diffusion metrics between in-vivo and ex-vivo tractography and no significant difference was found
	*In-vivo*	Siemens Healthcare, Prisma, 3T	SE-EPI	DTI	(Wang et al., 2023): Studied the mechanical properties of brain development
Camel	*Ex-vivo*	Philips Healthcare, Achieva, 3T	SE-EPI	DTI	(Cartiaux et al., 2023): Reconstructed the cingulum, corpus callosum, and internal capsule tracts in the one-humped camel brain.
Dolphin	*Ex-vivo*	Siemens Healthcare, Trio, 3T	DW-SSFP	DTI	(Berns et al., 2015): Determined the existence of a parietal auditory cortex area in the bottlenose dolphin
	*Ex-vivo*	General Electric Healthcare, 3T	SE-EPI	DTI	(Wright et al., 2018): Segmented the main white matter bundles in the bottlenose dolphin brain and studied their asymmetries
	*Ex-vivo*	Siemens Healthcare, Magnetom, 7T	DW-SSFP	DTI	(Orekhova et al., 2022): Studied the auditory pathway in the bottlenose dolphin
	*Ex-vivo*	Philips Healthcare, Achieva, 3T	SE-EPI	CSD	(Gerussi et al., 2023, 2024): Determined the prefrontal cortex position in the bottlenose dolphin based on thalamic connections.

## Discussion

MRI has established itself as an essential non-invasive technique in both neuroscientific research and clinical treatment (Assaf et al., 2019). The following development of DWI has expanded our understanding by digging into the microscopic characteristics of the white matter, allowing for the analysis of brain connections in both pathological and purely anatomico-physiological contexts using sophisticated tractography methods (Le Bihan, 1996). Clinical applications of DWI have not only transformed human medicine but have also proven beneficial in veterinary medicine, offering critical insights for neuropathological diagnoses in domestic animals (Hasegawa et al., 2003; Salma, 2015; Crawford et al., 2023). Furthermore, DWI has been effectively applied to domestic species often used in translational medicine, such as sheep and pigs, mostly demonstrating feasibility and setting standards for these species (Lee et al., 2015; Ella et al., 2019).

DWI, and consequently tractography, have merged as critical techniques for studying brain connections in large animals, including domestic and “wild” mammals such as dolphins or camels. Notably, DWI’s capacity to work in formalin-fixed tissues has broadened its application (D’Arceuil et al., 2007; Berns et al., 2015; Cook et al., 2018; Wright et al., 2018; Cartiaux et al., 2023; Gerussi et al., 2023, 2024; Graïc et al., 2023). Conducting invasive experiments and consequent euthanasia on these large, wild species are ethically nearly infeasible today. Furthermore, for the same reasons along with practical restrictions, potential *in-vivo* MRI in large species are extremely rare, and so far have not been published. As a result, *ex-vivo* imaging has emerged as the principal method for investigating the basis of complex cognitive abilities and behaviors in these species, providing outstanding neuroanatomical knowledge. *Ex-vivo* DWI also allows for significantly higher voxel resolutions maintaining a good SNR through adequate sequences such as STEM or SSFP, or re-hydration of the tissue with PBS, reaching scales of hundreds of micrometers. This level of resolution notably overcomes the limits of time-constrained *in-vivo* scans, allowing for an accurate mapping of brain structures and neural pathways.

This MRI technique has great potential for studying not only rare and endangered species but also extinct species whose unique specimens are currently fixed and preserved in museums or banks. Leveraging this option might allow the neuroscientific and broader scientific communities to investigate the evolution and development of brains across a wider range of taxa.

However, for DWI to give valid results, some factors must be considered. The first, and most important, is tissue fixation. There is no doubt that white matter integrity is central to any tract tracing algorithm (Beaulieu, 2002). In fact, the process of tissue fixation must happen as soon and swiftly as possible in order to block the autolytic mechanisms of the tissues which leads to the loss of integrity of the white matter. Fixation is a challenge and may not always be optimal in large animals for several factors such as the time between the death of the animal and awareness of park personnel (depending on the park, from minutes to several hours), the complexity of the vascular system supplying the brain (e.g., in cetaceans), the complex anatomy of the skull (such as that of rhinoceroses, elephants or giraffes) or ambient temperature (fast cooling of tissue seems to play a critical role in nervous tissue autolysis). All these factors are usually understood as a whole under the umbrella term of PMI, although wide variations can occur (e.g., a 24 h at 35°C ambient temperature is different in outcome from 24 h at 5°C), although a recent study seems to indicate minimal immunohistochemical effects (Koehler et al., 2024), and in details, PMI explains only part of the problems encountered in large brain fixation. For all these reasons, fixation by immersion seems to remain the most used alternative, as is the case in human brain preservation (Nardi et al., 2023). The fixative should be changed after the first 24 h, and a large volume is necessary (ideally 10 times the volume to be fixed). A topic still often discussed with confusion regards the type of fixative used. Finally, even after extraction, the size of some brains reaches ∼30 cm in diameter, and adapted coils may not exist.

The second main aspect to consider is the choice of DWI parameters. DTI, CSD, and other algorithms offer different approaches to analyze white matter diffusion anisotropy. Sequences used for DTI are fast, and do not require a high *b*-value or a high number of directions; however, DTI provides only a single 3D orientation per voxel and is, by definition, limited in the detection of multiple fiber populations within a voxel. On the other hand, sequences used for CSD require a long scan time with higher, multishelled *b*-values in a high number of directions. Comparatively, the CSD algorithm is able to identify multiple fiber orientations within a voxel without losing information, making it more robust for mapping complex fiber tracts. However, the DWI methods used for analysis must align with the research aims and objectives, fulfilling the specific requirements and questions posed by researchers, whether from a physics-based perspective or a biophysical-based perspective.

In conclusion, DWI and tractography have emerged as highly promising non-invasive MRI techniques for studying large animal brains. They offer remarkable insights into their complex and relatively understudied neuroanatomy and connectivity. Despite the challenges, drawbacks, and constraints, these tools represent a valuable addition to the limited toolbox available for enhancing our understanding of comparative brain anatomy, connections, and evolution.

## Author contributions

MB: Conceptualization, Funding acquisition, Writing – original draft, Writing – review & editing. J-MG: Conceptualization, Writing – original draft, Writing – review & editing. TG: Conceptualization, Visualization, Writing – original draft, Writing – review & editing.

## References

[B1] Anaya García M. S. Hernández Anaya J. S. Marrufo Meléndez O. Velázquez Ramírez J. L. Palacios Aguiar R. (2015). In vivo study of cerebral white matter in the dog using diffusion tensor tractography. *Vet. Radiol. Ultrasound* 56 188–195. 10.1111/vru.12211 25288360 PMC4409102

[B2] Andics A. Miklósi Á (2018). Neural processes of vocal social perception: Dog-human comparative fMRI studies. *Neurosci. Biobehav. Rev.* 85 54–64. 10.1016/J.NEUBIOREV.2017.11.017 29287629

[B3] Andrews E. F. Pascalau R. Horowitz A. Lawrence G. M. Johnson P. J. (2022). Extensive connections of the canine olfactory pathway revealed by tractography and dissection. *J. Neurosci.* 42 6392–6407. 10.1523/JNEUROSCI.2355-21.2022 35817576 PMC9398547

[B4] Assaf Y. Basser P. J. (2005). Composite hindered and restricted model of diffusion (CHARMED) MR imaging of the human brain. *Neuroimage* 27 48–58. 10.1016/j.neuroimage.2005.03.042 15979342

[B5] Assaf Y. Johansen-Berg H. Thiebaut de Schotten M. (2019). The role of diffusion MRI in neuroscience. *NMR Biomed.* 32:62. 10.1002/nbm.3762 28696013

[B6] Baliyan V. Das C. J. Sharma R. Gupta A. K. (2016). Diffusion weighted imaging: Technique and applications. *World J. Radiol.* 8:785. 10.4329/wjr.v8.i9.785 27721941 PMC5039674

[B7] Banstola A. Reynolds J. N. J. (2022). Mapping sheep to human brain: The need for a sheep brain atlas. *Front. Vet. Sci.* 9:961413. 10.3389/fvets.2022.961413 35967997 PMC9372442

[B8] Basser P. J. (1995). Inferring microstructural features and the physiological state of tissues from diffusion-weighted images. *NMR Biomed.* 8 333–344. 10.1002/nbm.1940080707 8739270

[B9] Basser P. J. Mattiello J. LeBihan D. (1994). MR diffusion tensor spectroscopy and imaging. *Biophys. J.* 66 259–267. 10.1016/S0006-3495(94)80775-1 8130344 PMC1275686

[B10] Basser P. J. Pajevic S. Pierpaoli C. Duda J. Aldroubi A. (2000). In vivo fiber tractography using DT-MRI data. *Magn. Reson. Med.* 44 625–632. 10.1002/1522-2594(200010)44:4<625::AID-MRM17>3.0.CO;2-O 11025519

[B11] Beaulieu C. (2002). The basis of anisotropic water diffusion in the nervous system – A technical review. *NMR Biomed.* 15 435–455. 10.1002/nbm.782 12489094

[B12] Behroozi M. Billings B. K. Helluy X. Manger P. R. Güntürkün O. Ströckens F. (2018). Functional MRI in the Nile crocodile: A new avenue for evolutionary neurobiology. *Proc. R. Soc. B Biol. Sci.* 285:20180178. 10.1098/rspb.2018.0178 29695446 PMC5936729

[B13] Behroozi M. Helluy X. Ströckens F. Gao M. Pusch R. Tabrik S. (2020). Event-related functional MRI of awake behaving pigeons at 7T. *Nat. Commun.* 11 1–12. 10.1038/s41467-020-18437-1 32948772 PMC7501281

[B14] Benjamin P. Khan F. Mackinnon A. D. (2017). The use of diffusion weighted imaging to evaluate pathology outside the brain parenchyma in neuroimaging studies. *Br. J. Radiol.* 90:821. 10.1259/bjr.20160821 28195506 PMC5605072

[B15] Berns G. S. Cook P. F. Foxley S. Jbabdi S. Miller K. L. Marino L. (2015). Diffusion tensor imaging of dolphin brains reveals direct auditory pathway to temporal lobe. *Proc. R. Soc. B Biol. Sci.* 282:20151203. 10.1098/rspb.2015.1203 26156774 PMC4528565

[B16] Billings B. K. Behroozi M. Helluy X. Bhagwandin A. Manger P. R. Güntürkün O. (2020). A three-dimensional digital atlas of the Nile crocodile (*Crocodylus niloticus*) forebrain. *Brain Struct. Funct.* 225:3. 10.1007/s00429-020-02028-3 32009190

[B17] Boucher S. Arribarat G. Cartiaux B. Lallemand E. A. Péran P. Deviers A. (2020). Diffusion tensor imaging tractography of white matter tracts in the equine brain. *Front. Vet. Sci.* 7:540253. 10.3389/fvets.2020.00382 32850994 PMC7406683

[B18] Cartiaux B. Amara A. Pailloux N. Paumier R. Malek A. Elmehatli K. (2023). Diffusion tensor imaging tractography in the one-humped camel (*Camelus dromedarius*) brain. *Front. Vet. Sci.* 10:1231421. 10.3389/fvets.2023.1231421 37649566 PMC10464492

[B19] Conrad M. S. Dilger R. N. Johnson R. W. (2012). Brain growth of the domestic pig (*Sus scrofa*) from 2 to 24 weeks of age: A longitudinal MRI study. *Dev. Neurosci.* 34 291–298. 10.1159/000339311 22777003 PMC3646377

[B20] Cook P. F. Berns G. (2022). Volumetric and connectivity assessment of the caudate nucleus in California sea lions and coyotes. *Anim. Cogn.* 25 1231–1240. 10.1007/s10071-022-01685-7 36114948

[B21] Cook P. F. Berns G. S. Colegrove K. Johnson S. Gulland F. (2018). Postmortem DTI reveals altered hippocampal connectivity in wild sea lions diagnosed with chronic toxicosis from algal exposure. *J. Comp. Neurol.* 526 216–228. 10.1002/cne.24317 28875534

[B22] Cook P. F. Hoard V. A. Dolui S. Frederick B. de B. Redfern R. (2021). An MRI protocol for anatomical and functional evaluation of the California sea lion brain. *J. Neurosci. Methods* 353:109097. 10.1016/j.jneumeth.2021.109097 33581216

[B23] Cozzi B. Bonfanti L. Canali E. Minero M. (2020). Brain waste: The neglect of animal brains. *Front. Neuroanat.* 14:573934. 10.3389/fnana.2020.573934 33304245 PMC7693423

[B24] Crawford A. H. Beltran E. Danciu C. G. Yaffy D. (2023). Clinical presentation, diagnosis, treatment, and outcome in 8 dogs and 2 cats with global hypoxic-ischemic brain injury (2010-2022). *J. Vet. Intern. Med.* 37 1428–1437. 10.1111/jvim.16790 37316975 PMC10365066

[B25] D’Arceuil H. E. Westmoreland S. de Crespigny A. J. (2007). An approach to high resolution diffusion tensor imaging in fixed primate brain. *Neuroimage* 35 553–565. 10.1016/j.neuroimage.2006.12.028 17292630

[B26] Dai G. Das A. Hayashi E. Chen Q. Takahashi E. (2016). Regional variation of white matter development in the cat brain revealed by ex vivo diffusion MR tractography. *Int. J. Dev. Neurosci.* 54 32–38. 10.1016/j.ijdevneu.2016.08.004 27568056 PMC5088495

[B27] Das A. Takahashi E. (2018). Characterization of white matter tracts by diffusion MR tractography in cat and ferret that have similar gyral patterns. *Cereb. Cortex* 28 1338–1347. 10.1093/cercor/bhx048 28334159 PMC6059242

[B28] Davis D. Ulatowski J. Eleff S. Izuta M. Mori S. Shungu D. (1994). Rapid monitoring of changes in water diffusion coefficients during reversible ischemia in cat and rat brain. *Magn. Reson. Med.* 31 454–460. 10.1002/mrm.1910310416 8208123

[B29] Dawe R. J. Bennett D. A. Schneider J. A. Vasireddi S. K. Arfanakis K. (2009). Postmortem MRI of human brain hemispheres: T2 relaxation times during formaldehyde fixation. *Magn. Reson. Med.* 61 810–818. 10.1002/mrm.21909 19189294 PMC2713761

[B30] De Santis S. Barazany D. Jones D. K. Assaf Y. (2016). Resolving relaxometry and diffusion properties within the same voxel in the presence of crossing fibres by combining inversion recovery and diffusion-weighted acquisitions. *Magn. Reson. Med.* 75 372–380. 10.1002/mrm.25644 25735538 PMC4737246

[B31] Dyrby T. B. Baaré W. F. C. Alexander D. C. Jelsing J. Garde E. Søgaard L. V. (2011). An ex vivo imaging pipeline for producing high-quality and high-resolution diffusion-weighted imaging datasets. *Hum. Brain Mapp.* 32 544–563. 10.1002/hbm.21043 20945352 PMC6870191

[B32] Ella A. Barrière D. A. Adriaensen H. Palmer D. N. Melzer T. R. Mitchell N. L. (2019). The development of brain magnetic resonance approaches in large animal models for preclinical research. *Anim. Front.* 9 44–51. 10.1093/af/vfz024 32002261 PMC6951960

[B33] Farquharson S. Tournier J. D. Calamante F. Fabinyi G. Schneider-Kolsky M. Jackson G. D. (2013). White matter fiber tractography: Why we need to move beyond DTI. *J. Neurosurg.* 118 1367–1377. 10.3171/2013.2.JNS121294 23540269

[B34] Förschler A. Boltze J. Waldmin D. Gille U. Zimmer C. (2007). MRI of experimental focal cerebral ischemia in sheep. *Rofo* 179 516–524. 10.1055/s-2007-962977 17436186

[B35] Foxley S. Jbabdi S. Clare S. Lam W. Ansorge O. Douaud G. (2014). Improving diffusion-weighted imaging of post-mortem human brains: SSFP at 7T. *Neuroimage* 102 579–589. 10.1016/j.neuroimage.2014.08.014 25128709 PMC4229505

[B36] Gage G. J. Kipke D. R. Shain W. (2012). Whole animal perfusion fixation for rodents. *J. Vis. Exp.* 65:3564. 10.3791/3564 22871843 PMC3476408

[B37] Garosi L. McConnell J. F. Platt S. R. Barone G. Baron J. C. de Lahunta A. (2006). Clinical and topographic magnetic resonance characteristics of suspected brain infarction in 40 dogs. *J. Vet. Intern. Med.* 20 311–321. 10.1111/j.1939-1676.2006.tb02862.x 16594588

[B38] Gerussi T. Graïc J. M. Cozzi B. Schlaffke L. Güntürkün O. Behroozi M. (2024). Constrained spherical deconvolution on diffusion-weighted images of dolphin brains. *Magn. Reson. Imaging* 108 104–110. 10.1016/j.mri.2024.02.002 38336113

[B39] Gerussi T. Graïc J. M. Grandis A. Peruffo A. Cozzi B. (2022). The orbitofrontal cortex of the sheep. Topography, organization, neurochemistry, digital tensor imaging and comparison with the chimpanzee and human. *Brain Struct. Funct.* 227 1871–1891. 10.1007/S00429-022-02479-W 35347401 PMC9098624

[B40] Gerussi T. Graïc J. M. Peruffo A. Behroozi M. Schlaffke L. Huggenberger S. (2023). The prefrontal cortex of the bottlenose dolphin (*Tursiops truncatus* Montagu, 1821): a tractography study and comparison with the human. *Brain Struct. Funct.* 228 1963–1976. 10.1007/s00429-023-02699-8 37660322 PMC10517040

[B41] Graïc J. M. Tagliavia C. Salamanca G. Gerussi T. Grandis A. Cozzi B. (2023). Connections of the sheep basolateral amygdala: A diffusion tensor imaging study. *J. Neurosci. Methods* 393:109883. 10.1016/j.jneumeth.2023.109883 37196786

[B42] Gray-Edwards H. L. Salibi N. Josephson E. M. Hudson J. A. Cox N. R. Randle A. N. (2014). High resolution MRI anatomy of the cat brain at 3 Tesla. *J. Neurosci. Methods* 227 10–17. 10.1016/J.JNEUMETH.2014.01.035 24525327 PMC4060963

[B43] Hakeem A. Y. Hof P. R. Sherwood C. C. Switzer R. C. Rasmussen L. E. L. Allman J. M. (2005). Brain of the African elephant (*Loxodonta africana*): Neuroanatomy from magnetic resonance images. *Anat. Rec. A Discov. Mol. Cell. Evol. Biol.* 287 1117–1127. 10.1002/ar.a.20255 16216009

[B44] Hamaide J. Lukacova K. Orije J. Keliris G. A. Verhoye M. van der Linden A. (2020). In vivo assessment of the neural substrate linked with vocal imitation accuracy. *Elife* 9:e49941. 10.7554/eLife.49941 32196456 PMC7083600

[B45] Hasegawa D. Orima H. Fujita M. Nakamura S. Takahashi K. Ohkubo S. (2003). Diffusion-weighted imaging in kainic acid-induced complex partial status epilepticus in dogs. *Brain Res.* 983 115–127. 10.1016/S0006-8993(03)03041-5 12914972

[B46] Jacqmot O. Van Thielen B. Fierens Y. Hammond M. Willekens I. Schuerbeek P. (2013). Diffusion tensor imaging of white matter tracts in the dog brain. *Anatom. Rec.* 296 340–349. 10.1002/ar.22638 23355519

[B47] Jacqmot O. Van Thielen B. Michotte A. de Mey J. Provyn S. Tresignie J. (2020). Neuroanatomical reconstruction of the canine visual pathway using diffusion tensor imaging. *Front. Neuroanat.* 14:558240. 10.3389/FNANA.2020.00054/BIBTEX PMC7461977 32973464

[B48] Jacqmot O. Van Thielen B. Michotte A. Willekens I. Verhelle F. Goossens P. (2017). Comparison of several white matter tracts in feline and canine brain by using magnetic resonance diffusion tensor imaging. *Anatom. Rec.* 300 1270–1289. 10.1002/ar.23579 28214332

[B49] Jelescu I. O. Palombo M. Bagnato F. Schilling K. G. (2020). Challenges for biophysical modeling of microstructure. *J. Neurosci. Methods* 344:108861. 10.1016/j.jneumeth.2020.108861 32692999 PMC10163379

[B50] Jeurissen B. Descoteaux M. Mori S. Leemans A. (2019). Diffusion MRI fiber tractography of the brain. *NMR Biomed.* 32:e3785. 10.1002/nbm.3785 28945294

[B51] Johnson P. J. Pascalau R. Luh W. M. Raj A. Cerda-Gonzalez S. Barry E. F. (2020). Stereotaxic diffusion tensor imaging white matter atlas for the in vivo domestic feline brain. *Front. Neuroanat.* 14:1. 10.3389/fnana.2020.00001 32116572 PMC7026623

[B52] Kang J. E. Lim M. M. Bateman R. J. Lee J. J. Smyth L. P. Cirrito J. R. (2009). Amyloid-β dynamics are regulated by orexin and the sleep-wake cycle. *Science* 326 1005–1007. 10.1126/science.1180962 19779148 PMC2789838

[B53] Kasukurthi R. Brenner M. J. Moore A. M. Moradzadeh A. Ray W. Z. Santosa K. B. (2009). Transcardial perfusion versus immersion fixation for assessment of peripheral nerve regeneration. *J. Neurosci. Methods* 184 303–309. 10.1016/J.JNEUMETH.2009.08.019 19723541

[B54] Knösche T. R. Anwander A. Liptrot M. Dyrby T. B. (2015). Validation of tractography: Comparison with manganese tracing. *Hum. Brain Mapp.* 36 4116–4134. 10.1002/hbm.22902 26178765 PMC5034837

[B55] Koehler J. W. Miller A. D. Rissi D. R. (2024). Effects of autolysis and prolonged formalin fixation on histomorphology and immunohistochemistry of normal canine brain tissue: An experimental study. *J. Vet. Diagn. Invest.* 36 169–176. 10.1177/10406387231220649 38212877 PMC10929626

[B56] Le Bihan D. (1996). *Diffusion, perfusion and functional mri: Functional MRI.* Milano: Springer, 23–27. 10.1007/978-88-470-2194-5_5

[B57] Lee W. Lee S. D. Park M. Y. Foley L. Purcell-Estabrook E. Kim H. (2015). Functional and diffusion tensor magnetic resonance imaging of the sheep brain. *BMC Vet. Res.* 11:1–8. 10.1186/s12917-015-0581-8 26467856 PMC4606502

[B58] Makada M. Matang M. (2023). Role of DWI in intracranial pathologies with its comparison to FLAIR and T2W imaging. *Int. J. Acad. Med. Pharm.* 5 433–437. 10.47009/jamp.2022.5.1.90

[B59] Mallon D. Dixon L. Campion T. Dawe G. Bhatia K. Kachramanoglou C. (2020). Beyond the brain: Extra-axial pathology on diffusion weighted imaging in neuroimaging. *J. Neurol. Sci.* 415:900. 10.1016/j.jns.2020.116900 32464349

[B60] Maranzano J. Dadar M. Bertrand-Grenier A. Frigon E. M. Pellerin J. Plante S. (2020). A novel ex vivo, in situ method to study the human brain through MRI and histology. *J. Neurosci. Methods* 345:108903. 10.1016/j.jneumeth.2020.108903 32777310

[B61] McConnell J. F. Garosi L. Platt S. R. (2005). Magnetic resonance imaging findings of presumed cerebellar cerebrovascular accident in twelve dogs. *Vet. Radiol. Ultrasound* 46 1–10. 10.1111/J.1740-8261.2005.00001.X 15693551

[B62] McFadden W. C. Walsh H. Richter F. Soudant C. Bryce C. H. Hof P. R. (2019). Perfusion fixation in brain banking: A systematic review. *Acta Neuropathol. Commun.* 7:146. 10.1186/s40478-019-0799-y 31488214 PMC6728946

[B63] McNab J. A. Miller K. L. (2010). Steady-state diffusion-weighted imaging: Theory, acquisition and analysis. *NMR Biomed* 23 781–793. 10.1002/nbm.1509 20886565

[B64] McNab J. A. Jbabdi S. Deoni S. C. L. Douaud G. Behrens T. E. J. Miller K. L. (2009). High resolution diffusion-weighted imaging in fixed human brain using diffusion-weighted steady state free precession. *Neuroimage* 46 775–785. 10.1016/j.neuroimage.2009.01.008 19344686

[B65] Merboldt K. D. Hänicke W. Frahm J. (1991). Diffusion imaging using stimulated echoes. *Magn. Reson. Med.* 19 233–239. 10.1002/mrm.1910190208 1881309

[B66] Miller K. L. McNab J. A. Jbabdi S. Douaud G. (2012). Diffusion tractography of post-mortem human brains: Optimization and comparison of spin echo and steady-state free precession techniques. *Neuroimage* 59 2284–2297. 10.1016/j.neuroimage.2011.09.054 22008372 PMC3314951

[B67] Moseley M. E. Kucharczyk J. Asgari H. S. Norman D. (1991). Anisotropy in diffusion-weighted MRI. *Magn. Reson. Med.* 19 321–326. 10.1002/mrm.1910190222 1652674

[B68] Mukherjee P. Berman J. I. Chung S. W. Hess C. P. Henry R. G. (2008). Diffusion tensor MR imaging and fiber tractography: Theoretic underpinnings. *Am. J. Neuroradiol.* 29 632–641. 10.3174/ajnr.A1051 18339720 PMC7978191

[B69] Nardi L. Schmeisser M. J. Schumann S. (2023). Fixation and staining methods for macroscopical investigation of the brain. *Front. Neuroanat.* 17:1200196. 10.3389/fnana.2023.1200196 37426902 PMC10323195

[B70] O’Connell A. B. Kuchel T. R. Perumal S. R. Sherwood V. Neumann D. Finnie J. W. (2021). Longitudinal magnetic resonance spectroscopy and diffusion tensor imaging in sheep (*Ovis aries*) with quinolinic acid lesions of the striatum: Time-dependent recovery of N-acetylaspartate and fractional anisotropy. *J. Neuropathol. Exp. Neurol.* 79 1084–1092. 10.1093/JNEN/NLAA053 32743645

[B71] Orekhova K. Selmanovic E. De Gasperi R. Gama Sosa M. A. Wicinski B. Maloney B. (2022). Multimodal assessment of bottlenose dolphin auditory nuclei using 7-tesla MRI, immunohistochemistry and stereology. *Vet. Sci.* 9:692. 10.3390/vetsci9120692 36548853 PMC9781543

[B72] Orije J. Cardon E. Hamaide J. Jonckers E. Darras V. M. Verhoye M. (2021). Uncovering a ‘sensitive window’ of multisensory and motor neuroplasticity in the cerebrum and cerebellum of male and female starlings. *Elife* 10:e66777. 10.7554/eLife.66777 34096502 PMC8219385

[B73] Peruffo A. Corain L. Bombardi C. Centelleghe C. Grisan E. Graïc J. M. (2019). The motor cortex of the sheep: Laminar organization, projections and diffusion tensor imaging of the intracranial pyramidal and extrapyramidal tracts. *Brain Struct. Funct.* 224 1933–1946. 10.1007/s00429-019-01885-x 31089853

[B74] Pfefferbaum A. Sullivan E. V. Adalsteinsson E. Garrick T. Harper C. (2004). Postmortem MR imaging of formalin-fixed human brain. *Neuroimage* 21 1585–1595. 10.1016/j.neuroimage.2003.11.024 15050582

[B75] Pieri V. Trovatelli M. Cadioli M. Zani D. D. Brizzola S. Ravasio G. (2019). In vivo diffusion tensor magnetic resonance tractography of the sheep brain: An atlas of the ovine white matter fiber bundles. *Front. Vet. Sci.* 6:485368. 10.3389/fvets.2019.00345 31681805 PMC6805705

[B76] Pirone A. Graïc J. M. Grisan E. Cozzi B. (2021). The claustrum of the sheep and its connections to the visual cortex. *J. Anat.* 238 1–12. 10.1111/joa.13302 32885430 PMC7755083

[B77] Rane S. Nair G. Duong T. Q. (2010). DTI at long diffusion time improves fiber tracking. *NMR Biomed.* 23 459–465. 10.1002/nbm.1482 20175137 PMC2949954

[B78] Robinson J. L. Baxi M. Katz J. S. Waggoner P. Beyers R. Morrison E. (2016). Characterization of structural connectivity of the default mode network in dogs using diffusion tensor imaging. *Sci. Rep.* 6 1–7. 10.1038/srep36851 27886204 PMC5122865

[B79] Roebroeck A. Miller K. L. Aggarwal M. (2019). Ex vivo diffusion MRI of the human brain: Technical challenges and recent advances. *NMR Biomed.* 32:e3941. 10.1002/nbm.3941 29863793 PMC6492287

[B80] Ronen I. Kim K. H. Garwood M. Ugurbil K. Kim D. S. (2003). Conventional DTI vs. slow and fast diffusion tensors in cat visual cortex. *Magn. Reson. Med.* 49 785–790. 10.1002/mrm.10431 12704758

[B81] Russo G. Helluy X. Behroozi M. Manahan-Vaughan D. (2021). Gradual restraint habituation for awake functional magnetic resonance imaging combined with a sparse imaging paradigm reduces motion artifacts and stress levels in rodents. *Front. Neurosci.* 15:805679. 10.3389/fnins.2021.805679 34992520 PMC8724036

[B82] Salma A. (2015). DTI in brain tumor surgery. *J. Neurosurg.* 122:474. 10.3171/2014.8.JNS141825 25526268

[B83] Schmidt M. J. Knemeyer C. Heinsen H. (2019). Neuroanatomy of the equine brain as revealed by high-field (3Tesla) magnetic-resonance-imaging. *PLoS One* 14:e0213814. 10.1371/journal.pone.0213814 30933986 PMC6443180

[B84] Schmidt M. J. Langen N. Klumpp S. Nasirimanesh F. Shirvanchi P. Ondreka N. (2012). A study of the comparative anatomy of the brain of domestic ruminants using magnetic resonance imaging. *Vet. J.* 191 85–93. 10.1016/j.tvjl.2010.12.026 21277239

[B85] Schmidt M. J. Pilatus U. Wigger A. Kramer M. Oelschläger H. A. (2009). Neuroanatomy of the calf brain as revealed by high-resolution magnetic resonance imaging. *J. Morphol.* 270 745–758. 10.1002/jmor.10717 19123244

[B86] Shaibani A. Khawar S. Shin W. Cashen T. A. Schirf B. Rohany M. (2006). First results in an MR imaging-compatible canine model of acute stroke. *Am. J. Neuroradiol.* 27 1788–1793. 16971637 PMC8139778

[B87] Shepherd T. M. Thelwall P. E. Stanisz G. J. Blackband S. J. (2009). Aldehyde fixative solutions alter the water relaxation and diffusion properties of nervous tissue. *Magn. Reson. Med.* 62 26–34. 10.1002/mrm.21977 19353660 PMC3188415

[B88] Straub S. Mangesius S. Emmerich J. Indelicato E. Nachbauer W. Degenhardt K. S. (2020). Toward quantitative neuroimaging biomarkers for Friedreich’s ataxia at 7 Tesla: Susceptibility mapping, diffusion imaging, R2 and R1 relaxometry. *J. Neurosci. Res.* 98 2219–2231. 10.1002/jnr.24701 32731306 PMC7590084

[B89] Takahashi E. Dai G. Rosen G. D. Wang R. Ohki K. Folkerth R. D. (2011). Developing neocortex organization and connectivity in cats revealed by direct correlation of diffusion tractography and histology. *Cereb. Cortex* 21 200–211. 10.1093/cercor/bhq084 20494968 PMC3025725

[B90] Takahashi E. Dai G. Wang R. Ohki K. Rosen G. D. Galaburda A. M. (2010). Development of cerebral fiber pathways in cats revealed by diffusion spectrum imaging. *Neuroimage* 49 1231–1240. 10.1016/j.neuroimage.2009.09.002 19747553 PMC2789885

[B91] Tournier J. D. Calamante F. Connelly A. (2007). Robust determination of the fibre orientation distribution in diffusion MRI: Non-negativity constrained super-resolved spherical deconvolution. *Neuroimage* 35 1459–1472. 10.1016/j.neuroimage.2007.02.016 17379540

[B92] Tournier J. D. Calamante F. Connelly A. (2012). MRtrix: Diffusion tractography in crossing fiber regions. *Int. J. Imaging Syst. Technol.* 22 53–66. 10.1002/ima.22005

[B93] Tovi M. Ericsson A. (1992). Measurements of T1 and T2 over time in formalin-fixed human whole-brain specimens. *Acta Radiol.* 33 400–404. 10.1177/028418519203300503 1389643

[B94] Tuch D. S. (2004). Q-ball imaging. *Magn. Reson. Med.* 52 1358–1372. 10.1002/mrm.20279 15562495

[B95] Walker M. R. Zhong J. Waspe A. C. Looi T. Piorkowska K. Drake J. M. (2019). Acute ex vivo changes in brain white matter diffusion tensor metrics. *PLoS One* 14:e0223211. 10.1371/journal.pone.0223211 31557265 PMC6762128

[B96] Wang S. Guertler C. A. Okamoto R. J. Johnson C. L. McGarry M. D. J. Bayly P. V. (2023). Mechanical stiffness and anisotropy measured by MRE during brain development in the minipig. *Neuroimage* 277:120234. 10.1016/j.neuroimage.2023.120234 37369255 PMC11081136

[B97] Wedeen V. J. Rosene D. L. Wang R. Dai G. Mortazavi F. Hagmann P. (2012). The geometric structure of the brain fiber pathways. *Science* 335 1628–1634. 10.1126/science.1215280 22461612 PMC3773464

[B98] Weiskopf N. Suckling J. Williams G. Correia M. Inkster B. Tait R. (2013). Quantitative multi-parameter mapping of R1, PD*, MT, and R2* at 3T: A multi-center validation. *Front. Neurosci.* 7:46379. 10.3389/fnins.2013.00095 23772204 PMC3677134

[B99] Werner M. Chott A. Fabiano A. Battifora H. (2000). Effect of formalin tissue fixation and processing on immunohistochemistry. *Am. J. Surg. Pathol.* 24 1016–1019. 10.1097/00000478-200007000-00014 10895825

[B100] Wright A. K. Theilmann R. J. Ridgway S. H. Scadeng M. (2018). Diffusion tractography reveals pervasive asymmetry of cerebral white matter tracts in the bottlenose dolphin (*Tursiops truncatus*). *Brain Struct. Funct.* 223 1697–1711. 10.1007/s00429-017-1525-9 29189908 PMC5884918

[B101] Yang S. Ghosh K. Sakaie K. Sahoo S. S. Ann Carr S. J. Tatsuoka C. (2019). A simplified crossing fiber model in diffusion weighted imaging. *Front. Neurosci.* 13:430469. 10.3389/fnins.2019.00492 31191215 PMC6541109

[B102] Zhang F. Daducci A. He Y. Schiavi S. Seguin C. Smith R. E. (2022). Quantitative mapping of the brain’s structural connectivity using diffusion MRI tractography: A review. *Neuroimage* 249:118870. 10.1016/j.neuroimage.2021.118870 34979249 PMC9257891

[B103] Zhang J. Aggarwal M. Mori S. (2012). Structural insights into the rodent CNS via diffusion tensor imaging. *Trends Neurosci.* 35 412–421. 10.1016/j.tins.2012.04.010 22651954 PMC3591520

[B104] Zhang H. Schneider T. Wheeler-Kingshott C. A. Alexander D. C. (2012). NODDI: Practical in vivo neurite orientation dispersion and density imaging of the human brain. *Neuroimage* 61 1000–1016. 10.1016/j.neuroimage.2012.03.072 22484410

[B105] Zhao F. Y. Kuroiwa T. Miyasakai N. Tanabe F. Nagaoka T. Akimoto H. (2006). Diffusion tensor feature in vasogenic brain edema in cats. *Acta Neurochir. Suppl.* 96 168–170. 10.1007/3-211-30714-1_37 16671448

